# Supplementary material for: Assessment of Nine Real-Time PCR Kits for African Swine Fever Virus Approved in Republic of Korea
Source: Viruses. 2024 Oct 17;16(10):1627. doi: 10.3390/v16101627 (PMC11512253; doi:10.3390/v16101627)
Supplement: Supplementary file 1 [file viruses-16-01627-s001.zip › viruses-3208148-supplementary/viruses-3208148-supplementary/Certificate of English editing.pdf]

editage

Editing Certificate

This document certifies that the manuscript listed below has been edited to ensure language and grammar accuracy and is error free in these aspects. The logical presentation of ideas and the structure of the paper were also checked during the editing process. The edit was performed by professional editors at Editage, a brand of Cactus Communications. The author's core research ideas were not altered in any way during the editing process. The quality of the edit has been guaranteed, with the assumption that our suggested changes have been accepted and the text has not been further altered without the knowledge of our editors.

MANUSCRIPT TITLE

Assessment of Nine Approved Diagnostic Kits in the Republic of Korea Based on Real-time PCR for African Swine Fever Vi-rus

AUTHORS

Siwon Lee, Tae Uk Han, Jin-Ho Kim

ISSUED ON

August 29, 2024

JOB CODE

THZZJ_3

THE EDITAGE Promise QUALITY GUARANTEE

Prabh Grewal
Senior Vice President - Editage

editage | helping you get published

Since 2002, Editage has helped over 430,000 authors publish around 1.2 million research papers in scholarly journals across over 1000 disciplines through editorial, translation, transcription, and publication support services. Editage is a brand of Cactus Communications (cactusglobal.com), a science communication and technology company.

CACTUS

GLOBAL :
+1(833) 979-0061 | request@editage.com

KOREA :
1533-6413 | submit-korea@editage.com
