# Supplementary material for: Assessment of Nine Real-Time PCR Kits for African Swine Fever Virus Approved in Republic of Korea
Source: Viruses. 2024 Oct 17;16(10):1627. doi: 10.3390/v16101627 (PMC11512253; doi:10.3390/v16101627)
Supplement: Supplementary file 1 [file viruses-16-01627-s001.zip › viruses-3208148-supplementary/viruses-3208148-supplementary/Supplementary figure 1 legend.pdf]

Supplementary Materials: Supplementary Figure S1: Comparison of analytical sensitivity of *African swine fever virus* (ASFV) real-time PCR kits using artificially spiked samples (ASSs).
